# Persistent high plasma levels of sCD163 and sCD14 in adult patients with measles virus infection

**DOI:** 10.1371/journal.pone.0198174

**Published:** 2018-05-24

**Authors:** Claudia Mascia, Irene Pozzetto, Blerta Kertusha, Raffaella Marocco, Cosmo Del Borgo, Tiziana Tieghi, Serena Vita, Stefano Savinelli, Marco Iannetta, Vincenzo Vullo, Miriam Lichtner, Claudio Maria Mastroianni

**Affiliations:** 1 Department of Public Health and Infectious Diseases, Sapienza University, Rome, Italy; 2 Infectious Diseases Unit, Sapienza University, S. M. Goretti Hospital, Latina, Italy; 3 National Institute for Infectious Diseases Lazzaro Spallanzani, IRCCS, Rome, Italy; Institut Cochin, FRANCE

## Abstract

**Background and aims:**

Measles is an infectious disease that represents a serious public health problem worldwide, being associated with increased susceptibility to secondary infections, especially in the respiratory and gastrointestinal tracts. The aim of this study was to evaluate sCD163 and sCD14 levels in measles virus (MV) infected patients, as markers of immune activation, in order to better understand their role in the pathogenesis of the disease. TNF-α plasma levels were also evaluated.

**Methods:**

sCD163, sCD14 and TNF-α were measured by ELISA in plasma samples of 27 MV infected patients and 27 healthy donors (HD) included as controls.

**Results:**

At the time of hospital admission, sCD163 and sCD14 levels were significantly higher in MV infected patients than in HD, while a decrease in TNF-α levels were found even if without statistical significance. sCD163 and sCD14 levels were significantly decreased after two months from acute infection compared to hospital admission although they remained significantly higher compared to HD. TNF-α levels increased significantly during the follow-up period. Considering clinical parameters, sCD163 levels positively correlated with aspartate aminotransferase, white blood cell count and neutrophils rate, while negatively correlated with the lymphocyte percentage. sCD14 levels positively correlated with the neutrophil and lymphocyte percentages.

**Conclusions:**

These results indicate that, despite the resolution of symptoms, an important macrophage/monocyte activation persists in measles patients, even after two months from infection.

## Introduction

Measles virus (MV) is one of the leading causes of death among young children globally, even though a safe and cost-effective vaccine is available. In 2015, there were 134.200 measles deaths globally, mostly children under the age of 5 years. Accelerated immunization activities have had a major impact in reducing measles deaths, which decreased by 79% from an estimated 651.600 in 2000 to 134.200 in 2015 [1]. In 2017, there were 14.393 cases in Europe and from December 2016 to November 2017, most of the cases were reported in Romania (41%), Italy (35%), Germany (7%) and Greece (4%) [2]. According to the Italian National Health Institute, there were 4885 measles cases in Italy from January 2017 to December 2017 [3].

Measles is a RNA virus of the genus *Morbillivirus*, subfamily *Paramyxovirinae* and family *Paramyxoviridae*. Measles is a highly contagious disease, transmitted through direct contact and the air. The virus infects the respiratory tract and then spreads throughout the body [4]. MV infection causes a severe but transient immunosuppression that can increase the risk of superinfections [5]. This immune depression may last for months and is characterized by a reduction in the number of lymphocytes both in children [6] and young adults [7]. A reduction in innate effector cells, including macrophages and natural killer cells has been described [8] with a marked decrease in T- and B-cells especially during the acute phase of the infection [9].

After viral clearance, lymphocyte counts tend to normalize within one week, while several reports showed a persistent effect on the immunological status with an impairment of non-measles memory cell repertoire [10]. Moreover, as recently reported, MV infection has been associated also with immune activation [11]. The response to viral infection is associated with elevated plasma levels of innate inflammatory cytokines such as tumor necrosis factor (TNF)-α, Interleukin (IL)-1β, IL-6, and IL-18, suggesting a potential role of innate immune response and type 1 immune activation in the pathogenesis of the disease [11].

In this study we investigated the activation of the immune system in a cohort of adult patients with measles virus infection, by assessing the longitudinal changes of plasma levels of soluble (s) CD163, sCD14, TNF-α at the time of hospitalization and during follow-up.

## Materials and methods

### Study population

The study population included a total of 27 patients with MV infection, confirmed by the detection of MV-specific immunoglobulin (Ig)-M antibody, admitted to the Infectious Disease Unit of the University of Rome “La Sapienza” in Latina. Twenty-seven healthy donors (HD) with similar age and sex distribution (32, 22–65 years; 40% male) were included as controls.

The study was approved by the local Ethics Committee CE Lazio 2 (N.0068451/2016). A written informed consent was obtained from all participants before enrolment in the study. All data were collected in the respect of study participants’ confidentiality and privacy.

### Clinical parameters

All patients underwent a biochemical assessment to determine white blood cell (WBC) and lymphocyte absolute count, the neutrophil and lymphocyte percentage, and platelet (PLT) absolute count, serum levels of aspartate aminotransferase (AST), alanine aminotransferase (ALT), gamma-glutamyl transferase (γGT), lactate dehydrogenase (LDH) and C-Reactive Protein (CRP). We also collected information on pulmonary, intestinal and liver impairment, and the severity of mucosal involvement.

### Measles IgM Ab testing

Measles specific IgM were assessed in all serum samples using a commercial measles IgM enzyme-linked immunosorbent assay kit (Architect Abbott).

### Samples handling

A total of 54 plasma samples were collected from MV infected patients and 27 from healthy donors. For each subject blood was collected in EDTA and heparin tubes following the manufacturer’s instructions, and after centrifugation plasma samples were frozen at -80° until use. Plasma were collected for all patients at the time of hospital admission (T0), for 20 subjects after 1 week (T1). For 7 patients, supplementary plasma samples were collected at 8 weeks of follow-up (T2).

### Detection of soluble biomarkers

Soluble markers of innate immune activation sCD163, sCD14 and TNF-α were detected with commercially available ELISA kits in duplicate (Quantikine human CD163 ELISA, Quantikine human CD14 ELISA, R&D Systems, Minneapolis, MN and LEGEND MAX™ Human TNF-α ELISA Kit, BioLegend, San Diego, CA) as described in detail previously [12]. The limits of detection for sCD163, sCD14 and TNF-α were 0.177 ng/ml, 0.125 ng/ml and 3.5 pg/ml respectively.

### Statistical analysis

GraphPad Prism Software version 5 (Software MacKiev) was used for statistical analysis. Values are given as median and ranges. Non-parametric Mann-Whitney test was performed to examine the differences between healthy controls and patients and non-parametric Wilcoxon test was used to evaluate repeated determinations of soluble markers in longitudinally collected samples. Spearman’s correlation coefficient (r) was calculated to analyze the correlations between inflammatory and clinical parameters. Finally, *p*-values less than <0.05 were considered significant.

## Results

### Demographic and clinical characteristics of study population

General and clinical characteristics of study subjects are listed in Table 1. No microbiologic evidence of other viral or bacterial infections was detected. All patients were of Italian origin. All patients were hospitalized and the median time of hospitalization was 7 days (range 4–12). None of the patients had previously been vaccinated against measles virus. Two patients were diabetic; 3 patients had hypertension; 22 subjects did not have any known comorbidities. The following complications were recorded: pneumonia (8/27), keratitis (1/27), diarrhoea (12/27), hepatitis (14/27). Moreover, 15 patients required oxygen supplementation and 6 (6/27) subjects were started on systemic corticosteroids after the first blood samples was collected.

**Table 1:** General and clinical information of study subjects.

Characteristics	MV patients n = (27)
**Age (Years)**	**30 (17–66)**
**Male/female; n (% male)**	**13/14 (48%)**
**Complications**	
**Diabetes**	**2 (7%)**
**Hypertension**	**3 (11%)**
**O**_**2**_ **Sat (%)**	**94 (89–98)**
**WGBs (10**^**3**^**/mL)**	**4.05 (2.3–8.9)**
**Lymphocytes (10**^**3**^**/mL)**	**0.82 (0.23–2.5)**
**% neutrophils**	**75 (33.9–93)**
**% lymphocytes**	**18.1 (5.6–52.3)**
**AST level, IU/L**	**102 (19–885)**
**ALT level, IU/L**	**206 (14–781)**
γ**GT level, IU/L**	**258 (18–766)**
**LDH level, IU/L**	**463 (213–923)**
**PLT (10**^**9**^**/L)**	**162 (55–286)**
**CRP**	**1.75 (0.45–12.26**

Results are expressed as median (range). MV: measles virus, HD: healthy donors, Sat: saturation, WGBs: white blood cells, AST: aspartate aminotransferase, ALT: alanine aminotransferase, γGT: gamma-glutamyl transferase, LDH: lactate dehydrogenase, PLT, platelets, CRP: C-Reactive Protein.

### Soluble biomarkers plasma levels at the time of hospital admission

**Fig 1:**
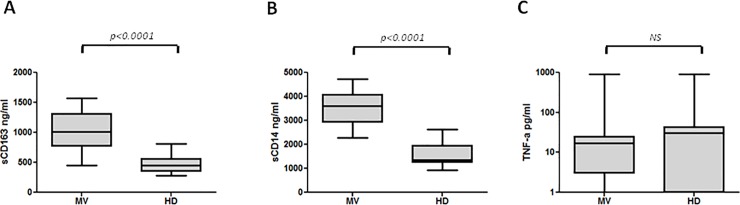
Plasma levels of sCD163, sCD14 and TNF-α in MV infected patients and healthy controls. (A) Box plots represent circulating levels of sCD163 in MV infected patients at T0 (n = 27) and in HD (n = 27). (B) Box plots represent circulating levels of sCD14 in MV infected patients at T0 (n = 27) and in HD (n = 27). (C) Box plots represent circulating levels of TNF-α in MV infected patients at T0 (n = 27) and in HD (n = 27). Horizontal bars represent the median values. For TNF-α graph a log10 scale was used. Statistical differences were assessed by the Mann-Whitney test. MV: measles virus; HD: healthy donors.

sCD163 plasma levels (median, ranges) were significantly higher in MV infected patients at hospital admission (T0) (1011.20, 456.21–1568.43) than in HD (453.4, 279.16–810.54) (p<0.0001) (Fig 1A). Similarly, sCD14 plasma levels were significantly higher at T0 in MV infected patients (3614, 2280–4736) compared to HD (1365.7, 917.4–2627.4) (p<0.0001) (Fig 1B). Conversely, regarding TNF-α plasma levels, lower values were found at T0 in MV infected patients (16.50, 0–905) than in HD (31, 0–909) but the difference was not statistically significant (p = 0.13) (Fig 1C).

### Soluble biomarkers plasma levels during the course of follow-up

**Fig 2:**
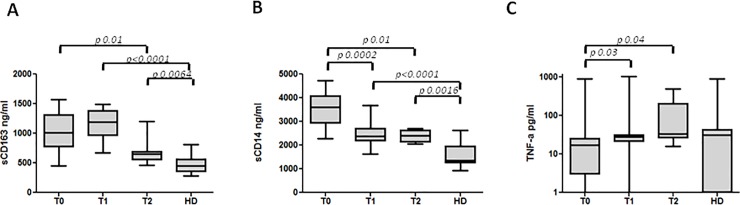
Longitudinal plasma levels of sCD163, sCD14 and TNF-α in MV infected patients and in HD. (A) Box plots represent circulating levels of sCD163 in MV infected patients at T0 (n = 27), T1 (n = 20), T2 (n = 7) and in HD (n = 27). (B) Box plots represent circulating levels of sCD14 in MV infected patients at T0 (n = 27), T1 (n = 20), T2 (n = 7) and in HD (n = 27). (C) Box plots represent circulating levels of TNF-α in MV infected patients at T0 (n = 27), T1 (n = 20), T2 (n = 7) and in HD (n = 27). Horizontal bars represent the median values. For TNF-α graph a log10 scale was used. Statistical differences were assessed by the Mann-Whitney and Wilcoxon tests. MV: measles virus; HD: healthy donors; T0: time of admission; T1: after 1 week; T2: 8 weeks of therapy.

sCD163 plasma levels were persistently higher at T1 (after one week) in MV patients (1191.63, 668–1490.72) (p<0.0001). Considering a subgroup of patients who consented to have follow-up blood samples taken after two months (T2) from the onset of symptoms (7 subjects), sCD163 levels were decreased compared to T0 (p<0.05), although they did not reach normal values and remained significantly higher than in HD (p = 0.0064) (Fig 2A). Furthermore, sCD14 plasma levels at T1 (2389, 1633–3688) were decreased compared to T0, although they remained persistently higher than HD (p<0.0001). At T2, considering the same subgroup of 7 patients, sCD14 levels were significantly lower compared to T0 (2392, 2042–2716) (p = 0.01), with no relevant differences compared to T1, but remained significantly higher than in HD (p = 0.0016) (Fig 2B). Regarding TNF-α plasma levels were significantly lower at T0 (16.50, 0–905) than T1 (28, 0–1035) and T2 (34, 16–505) (p = 0.03 and p = 0.04 respectively) (Fig 2C).

### Soluble markers, demographic and clinical parameters

Considering demographic and clinical parameters sCD163 levels positively correlated with aspartate transaminase (AST) values at T0 (r = 0.5; p = 0.009), suggesting an association with liver damage. After stratifying patients according to alanine transaminase (ALT) levels into two groups, one with elevated ALT and one with normal ALT, the first group had significantly higher sCD163 levels, compared to the latter (p = 0.013). Considering TNF-α plasma levels, a strong positive correlation with CRP values was found (r = 0.7; p = 0.006). Moreover, we found higher sCD163 levels in patients with diarrhoea and higher TNF-α levels in patients with respiratory failure, although this findings did not reach the statistical significance. No differences were found in other clinical parameters, such as exanthema extension. A positive correlation between sCD14 levels and the rate of both T-lymphocytes (r = 0.4; p = 0.01) and neutrophils (r = 0.4; p = 0.01) was found. Furthermore, higher levels of sCD14 levels were found in patients with respiratory failure, even though this finding did not reach the statistical significance. Considering demographic parameters, no significant differences were found between men and women, and between patients older and younger than 30 years. No changes in the results were observed after the exclusion of 5 patients with comorbidities, suggesting the absence of a contribution of comorbidities in changes in inflammatory markers. An accurate statistical analysis was also performed to evaluate the influence of steroid use on sCD63 plasma levels in the longitudinal evaluation: after exclusion of 6 patients who were treated with steroids, no changes in the results were observed (S1 Fig). All correlations between soluble biomarkers and clinical parameters are listed in Table 2.

**Table 2:** Correlations between clinical and immunological parameters.

Variable	sCD163	sCD14	TNF-α
**Age**			
**r**	**0.02**	**-0.04**	**0.1**
***P***	**0.9**	**0.83**	**0.59**
**WGBs**			
**r**	**0.25**	**-0.02**	**-0.3**
***P***	**0.2**	**0.9**	**0.07**
**AST**			
**r**	**0.5**	**0.27**	**-0.3**
***P***	**0.009**	**0.18**	**0.12**
**ALT**			
**r**	**0.36**	**0.25**	**-0.3**
***P***	**0.07**	**0.21**	**0.15**
**γGT**			
**r**	**0.37**	**0.29**	**-0.04**
***P***	**0.058**	**0.14**	**0.86**
**LDH**			
**r**	**0.28**	**0.34**	**-0.23**
***P***	**0.16**	**0.088**	**0.27**
**Lymphocytes**			
**r**	**-0.2**	**-0.3**	**0.07**
***P***	**0.19**	**0.078**	**0.7**
**% neutrophils**			
**r**	**0.29**	**0.4**	**-0.06**
***P***	**0.13**	**0.01**	**0.7**
**% lymphocytes**			
**r**	**-0.3**	**-0.4**	**0.13**
***P***	**0.13**	**0.01**	**0.5**
**PLT**			
**r**	**0.21**	**-0.2**	**-0.17**
***P***	**0.29**	**0.22**	**0.40**
**CRP**			
**r**	**-0.3**	**0.2**	**0.7**
***P***	**0.34**	**0.47**	**0.006**

Spearman’s correlation coefficients (*r*) and level of statistical significance (*P*) are indicated. WGBs: white blood cells, AST: aspartate aminotransferase, ALT: alanine aminotransferase, GγGT: gamma-glutamyl transferase, LDH: lactate dehydrogenase, PLT: platelets, CRP: C-Reactive Protein.

## Discussion

In the present study, we assessed monocytes/macrophages activation biomarkers in adult patients with MV infection by evaluating plasma levels of sCD163, sCD14 and TNF-α during both the acute and recovery stages of the infection. Our data showed that MV infection is associated with an increase in inflammatory biomarkers in adult patients, which persists for several weeks after recovery from the disease. Several studies described a condition of immune suppression, immediately after MV infection, which represents a major cause of increased risk of secondary infections and complications. During the acute phase of measles infection, lymphocytes can show phenotypic and numeric alterations with increased susceptibility to apoptosis [13]. Specifically, circulating blood leukocytes are more susceptible to apoptosis because of an increase in the expression of Fas and subsequent binding of annexin-V. Acute phase is followed by a recovery phase in which a rapid normalization of cell counts is usually experienced [6].

During the acute phase of MV infection, the peak of viremia is associated with a marked reduction of T- and B-cell counts, as showed by de Vries RD et al. [9]. The subsequent expansion of specific cell-mediated and humoral response leads to the control of MV replication and the resolution of the disease [14]. However, the restoration of normal lymphocyte cell counts does not coincide with a complete normalization of the immunological responsiveness. Some observations showed a reduced capacity to respond to other pathogens, up to 3 years after MV infection, thus suggesting T cell functional impairment [15]. Conversely, high T proliferative responses have been documented in vivo [9]. An “immune amnesia” has been proposed by De Vries et al. [9] to explain the simultaneous immune suppression and immune activation induced by MV, with loss of memory T cell specific for non-measles pathogens and expansion of measles specific T lymphocytes. Interestingly, a higher mortality over a period of three years after MV infection has been described in children, suggesting a persistent effect on the immune system [16]. Recently a pivotal role of macrophage and innate immunity has been described in children in the context of acute MV infection, during which immunosuppression is accompanied by a cytokine storm [11]. The main cellular receptor for MV has been identified in CD150 (also known as SLAMF1), which is expressed on B- and T-lymphocytes, dendritic cells and monocytes [17–19]. Alveolar macrophages and CD11c+ dendritic cells seem to be the “early target cells” [20–22].

The first result of our study was the increased production of sCD163 and sCD14 during the acute phase of MV infection in patients compared to healthy donors, indicating an activation of monocyte/macrophage system. CD163 is an important surface marker expressed on monocyte-macrophage lineage cells and shed in the soluble form during inflammation. Recently increased plasma levels of sCD163 have been found in several infections (HIV, HIV/CMV coinfection, hepatitis, sepsis, tuberculosis) and have been associated with higher risk of inflammation and organ diseases, such as diabetes, obesity, atherosclerosis and liver impairment [12,23–27]. In our study, we found higher levels of sCD163 in MV infected patients at hospital admission compared to HD, suggesting that immune activation was present in parallel with immune suppression. After one week, we observed persistently elevated plasma levels, while after two months sCD163 levels significantly decreased in a subgroup of 7 patients compared to T0, but remained significantly higher than in HD. As suggested by Esolen LM et al., monocyte/macrophages represent primary target cells for MV during the acute phase of the infection in humans [28]. Indoh T et al. observed that human monocytic cell lines are persistently infected with measles virus and their ability of producing cytokines after lipopolysaccharide (LPS)-stimulation was significantly impaired [29].

Similar results were obtained for sCD14, the soluble form of a glycosyl phosphatidyl inositol, which is expressed mainly on monocytes, macrophages and dendritic cells and is released upon monocyte activation. In our study population, we found a significant increase of sCD14 in MV patients at the time of hospital admission compared to HD. Conversely sCD14 plasma levels at T1 decreased significantly compared to T0 but remained significantly higher than in HD. After two months sCD14 levels were significantly decreased compared to T0 in the same subgroup of 7 patients as previously described for sCD163. Despite a decrease in concentrations, sCD14 levels did not reach normal values and remained significantly higher than in HD. These results reflect a severe and long lasting immune activation. Recently, the evolution of T cell responses in MV-infected rhesus macaques was monitored for a period of six months and a prolonged presence of viral RNA in PBMC was observed [30]. These data confirmed previous reports showing a delayed clearance of MV-RNA in both naturally infected children [31] and rhesus macaques [32]. MV infects several cell types, such as lymphocytes, monocyte/macrophages, epithelial cells and endothelial cells [28,33]. sCD14 and sCD163 are associated with the innate immune response and are produced following the infection of target cells, such as monocytes and tissue macrophages. In our study the persistently incresed levels of soluble factors of monocyte/macrophage activation could be linked to a persistent exposure to MV, secondary to a delayed viral clearance, with prolonged activation of these cell subsets.

In addition, the intestine represents an important site of MV persistence, with relevant antigenic stimulation, as suggested by Lewin J et al. [34]. MV persistence in the gut could lead to deterioration of the mucosal barrier integrity with subsequent bacterial translocation. In this context, bacterial translocation could explain the significant and persistent immune activation of monocyte/macrophages, leading to long lasting release of soluble factors. Moreover, several chronic diseases have been linked with MV persistence in target tissues, such as Crohn's disease [34–35].

In our study, we did not find a statistically significant differences in TNF-α plasma levels in MV infected patients at hospital admission compared to HD. Conversely, after one week, and after two months an increase in TNF-α plasma levels was observed, suggesting a recovery of TNF-α production after MV infection, whereas sCD163 and sCD14 persisted increased. Interestingly, Leopardi et al. showed a reduced production of TNF-α by both MV infected peripheral blood monocytes and human monocytic THP-1 cell line [36]. Despite this in vitro evidence, in vivo data are limited and are available only for children. Lin et al. showed higher TNF-α plasma levels in MV infected children with deadly disease or HIV-infection [11]. These data cannot be directly compared with our data, obtained from a cohort of adults, in which plasma TNF-α seems to play a marginal role.

The limitations of the study are the lack of MV-RNA detection and quantification, the small number of plasma samples available at 8 weeks of follow-up, and the relatively short observation time after acute measles infection. Moreover, almost all patients presented complicated MV infection, limiting the possibility to find significant differences between complicated and uncomplicated disease.

In conclusion, our results suggest that MV infection is associated with marked and persistent monocyte/macrophage activation, characterized by increased sCD163 and sCD14 levels in plasma, even several weeks after disease recovery. A longer follow-up and a wider study population are needed in order to define how long the immune activation state persists and better understand the clinical significance of soluble markers elevation, which could be employed for identifying those patients with an enhanced risk of developing long term complications.

## Supporting information

S1 Fig Longitudinal plasma levels of sCD163 in all MV infected patients and in MV infected patients excluding patients treated with steroids. (A) Box plots represent circulating levels of sCD163 in all MV infected patients at T0 (n = 27), T1 (n = 20), T2 (n = 7) and in HD (n = 27). (B) Box plots represent circulating levels of sCD163 in MV infected patients excluding patients who were treated with steroids at T0 (n = 21), T1 (n = 16), T2 (n = 5) and in HD (n = 27). Horizontal bars represent the median values. Statistical differences were assessed by Mann-Whitney. T0: time of admission; T1: after 1 week; T2: 8 weeks of therapy; HD: healthy donors.
(TIF)

## Abbreviations

MV: measles virus
WBCs: white blood cells
γGT: gamma-glutamyl transferase
LDH: lactate dehydrogenase
PLT: platelets
AST: aspartate aminotransferase
ALT: alanine aminotransferase
HD: healthy donors
sCD14: soluble CD14
sCD163: soluble CD163
TNFα: tumor necrosis factor α
