# Supplementary figures and images for: Persistent high plasma levels of sCD163 and sCD14 in adult patients with measles virus infection
Source: PLoS One. 2018 May 24;13(5):e0198174. doi: 10.1371/journal.pone.0198174 (PMC5967820; doi:10.1371/journal.pone.0198174)

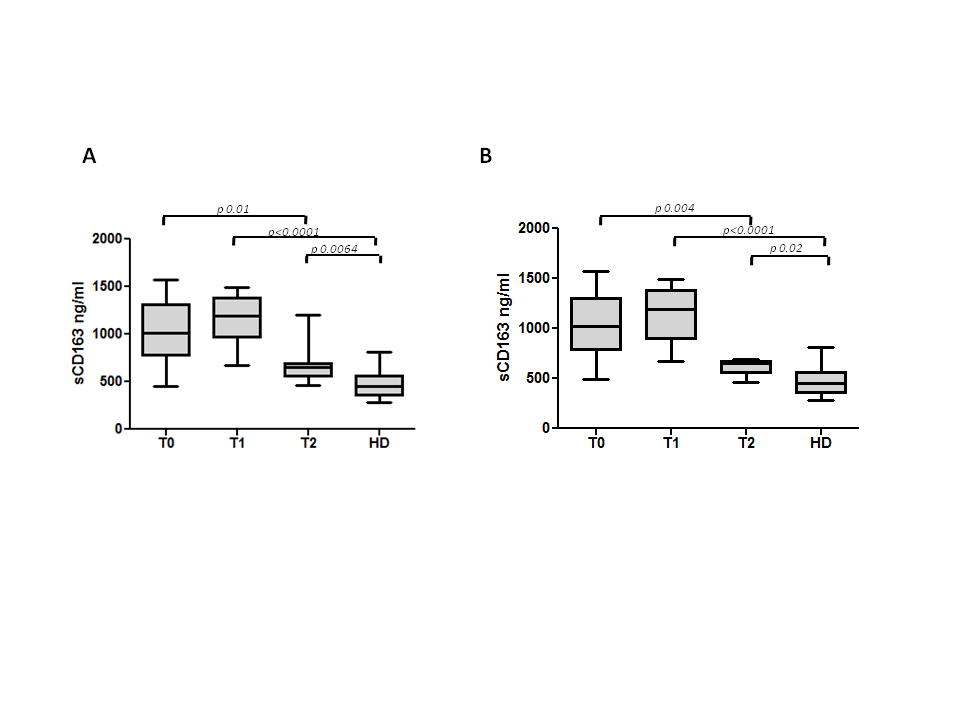

Supplement: S1 Fig — (A) Box plots represent circulating levels of sCD163 in all MV infected patients at T0 (n = 27), T1 (n = 20), T2 (n = 7) and in HD (n = 27). (B) Box plots represent circulating levels of sCD163 in MV infected patients excluding patients who were treated with steroids at T0 (n = 21), T1 (n = 16), T2 (n = 5) and in HD (n = 27). Horizontal bars represent the median values. Statistical differences were assessed by Mann-Whitney. T0: time of admission; T1: after 1 week; T2: 8 weeks of therapy; HD: healthy donors. (TIF) [file pone.0198174.s001.tif]
